# Does intellectual capital efficiency measured by modified value-added intellectual coefficient affect the financial performance of insurance companies in Ethiopia?

**DOI:** 10.1371/journal.pone.0295321

**Published:** 2024-01-19

**Authors:** Samuel Godadaw Ayinaddis, Habtamu Getachew Tegegne, Nitsuh Alemayehu Belay

**Affiliations:** 1 Department of Management, Woldia University, Woldia, Amhara, Ethiopia; 2 Department of Accounting and Finance, Woldia University, Woldia, Amhara, Ethiopia; Babes-Bolyai University, Cluj-Napoca, ROMANIA

## Abstract

Insurance company managers and shareholders should be conscious of the effect of intellectual capital efficiency and its components on financial performance. *The purpose of this study is to examine the role of intellectual capital efficiency and its components on the financial performance of insurance companies*. *To achieve study objectives Modified value-added intellectual coefficient is adopted to measure the effect of intellectual capital efficiency*. *The study adopted an explanatory research design with an arrangement of secondary data analysis via document analysis*, *quantitative approach*, *and deductive method of inquiry*. *Panel data was used with a sample of 14 insurance companies from 2012–2022*. *Descriptive and regression analyses were performed to analyze the data using STATA version 15*.*0*. *Econometric model estimation procedures and multiple regression assumptions were tested accordingly*. *The random effect regression result revealed that the value-added intellectual capital and its component human capital and capital employed efficiency have a positive significance association with financial performance*. *Whereas*, *relational capital efficiency and structural capital efficiency do not have a significant contribution to the financial performance of insurance sectors in Ethiopia*. The findings of this study contribute to the theoretical and practical understanding of the relationship between intellectual capital efficiency and financial performance in the context of insurance companies in Ethiopia.

## Introduction

Traditionally, the success of economic development greatly depends on the use of tangible assets like natural resources, land, and equipment [1]. According to the traditional resource-based view of the firm, certain types of resources (both physical and human) owned by the organization have the potential and promise to generate competitive advantage, which ultimately leads to superior organizational performance [2, 3].

Intellectual capital is a relatively recent concept [4]. It is acknowledged as the new invisible asset [5]. It is a superior resource for creating economic wealth [6] and a crucial component of running a flourishing business [7]. Intellectual capital has been considered an inalienable and indispensable resource predominantly in environments where the source of competitive advantage is strongly based on knowledge and intangible resources [8]. IC becomes the heart of knowledge-based growth [9]. This shows knowledge is a pivotal enabler for sustainable economic growth and social development. It is a part of intellectual capital which used as a more competitive advantage for service delivery industries like insurance companies.

In the current knowledge economy, intellectual capital builds up its recognition due to its important feature for sustainable organizational performance [10]. On the other hand, traditional base sources of competitive advantage depend on tangible assets in creating firm value and sustaining competitive advantage begun to erode [11]. The survival and competitive success of organizations depends on the strategic management of intangible assets(IC) as compared to physical resources [5].

According to the traditional resource-based view, internal resources are the strength of the firm (3), if they possess the characteristics of rare, valuable not substitutable, and imperfectly imitable (VRIN) [2, 12]. A firm’s competitiveness and financial performance are achieved by long-lasting heterogeneity of resources [13]. This theory addresses how firms achieve sustainable competitive advantage by deploying their resources [14]. However, the dynamic capability theory is derived from RBV theory to compensate for the shortcomings of RBV on how sustainable competitive advantage and superior performance in achieved in the dynamic environment [15]. This theory stresses that even if firms possess VRIN resources but do not deploy dynamic capabilities, the firms’ competitiveness, superior return, and financial performance may be short-lived if the environment is changed [10]. Further, the knowledge-based view of the firm argues that success, superior return financial performance, and competitiveness are not based much on tangible resources rather it is based on intangible knowledge capabilities [16]. Knowledge is a fundamental and key input for production and the only means of sustainable superior firm’s performance, which is difficult to imitate [17].

Furthermore, the theory of intellectual capital states that intangible assets are vital components of firm value [18] Thisich includes human capital (knowledge, talent, ability, invention, and skill of employees) structural capital (intellectual assets which would be created by the firms like systems, procedures, databases, copyrights and patents and capital employed includes both physical and financial asset of firms [19]. Investing in intellectual capital is an effective mechanism for enhancing competitiveness and value creation [19].

A study conducted by [20], acknowledged that the banking sector relied more on IC followed by insurance companies and Brokerage firms. [21] suggested that intellectual capital efficiency has a significant contribution to both life and non-life insurance companies. Whereas, [22], stated that IC has a higher impact on the corporate performance of the Information technology industry than other industries. In addition to this. [23–25] States that IC has a positive and significant impact on the financial performance of insurance companies. Further, [26]) Revealed that to improve their financial performance insurance companies should increase intellectual capital.

However, a study conducted by [27, 28] revealed that IC did not weigh upon business performance and does not have an insignificant impact on financial performance, but HCE has a significant impact among the components of intellectual capital. In Ethiopia, a study carried out in the banking industry by [29] stated that ICE and CEE have a positive significant impact on the financial performance and productivity of Ethiopian commercial banks. In addition to this, HCE has no significant effect on ROE. In the same vein, research conducted by [30] suggested ICE, HCE, and SCE have a positive significant impact on the financial performance of banks in Ethiopia.

In Ethiopia conducting this study was demanded, due to different theoretical views and empirical studies contradicting each other. In addition, most previous studies concerned with the relationship between intellectual capital efficiency and financial performance have been carried out in the banking industry [29]. The other research gap that motivated the researchers to conduct this study was, to the best of the researcher’s knowledge, there exists no empirical study conducted in Ethiopia on the effect of intellectual capital efficiency on the financial performance of insurance sectors. Hence, this study examines the effect of intellectual capital measured with a modified value-added intellectual coefficient (MVAIC^™^) and its components (HCE, RCE, SCE & CEE) on the financial performance of insurance sectors measured by ROA. Therefore, with controversial theoretical and empirical reviews policy makers, insurance company managers, and shareholders should be conscious about the effect of intellectual capital efficiency and its components on financial performance.

The remaining sections are organized as follows: the *Literature* section provides an overview of the existing literature; the *Research Methodology* section discusses the research methodology, including the research design, target population, sampling design, sources of data and data collection instruments, and the measurement of variables and study analysis; the *Data Presentation and Discussions* present the findings and the last section presents the *Conclusions*

## Objective of the study

### General objective

The main objective of this study is to investigate the effect of intellectual capital efficiency on the financial performance of insurance sectors in Ethiopia.

### Specific objectives

Based on the major objective of the study, the following specific objectives are drawn.

1. To examine each insurance company’s intellectual capital value creation efficiency using modified value-added intellectual coefficient (MVAIC^™^) measurement.
2. To examine the components of modified intellectual capital efficiency (Human capital efficiency, relation capital efficiency, structural capital efficiency, and capital employed efficiency) on the financial performance of insurance sectors in Ethiopia.

## Hypothesis of the study

**Ho** intellectual capital efficiency and its components do not have a significant impact on the financial performance of insurance companies in Ethiopia.

## Review related literature

### Empirical reviews

An empirical study conducted by [26] on 9 publicly listed Indonesian insurance sectors from the period 2009–2013 using Granger causal analysis revealed that the dominant influence on the financial performance of intellectual capital rather than intellectual capital on financial performance. Another study by [31] on the role of intellectual capital efficiency on financial performance using MVAIC^™^ on a firm’s performance on Korean manufacturing company from the period 2013–2018 concluded that physical capital is the most influential factor for a firm’s financial performance, HCE has positive impact While SCE hurt profitability of Korean manufacturing firms. Further, the study suggested that MVAIC is a more accurate measure than Public 1998 VAIC^™^ efficiency.

A study by [21] on Pakistan insurance companies from the study period 2006–2010 on the role of VAIC efficiency on financial performance states that VA and VAIC^™^ have a positive relation with financial performance. HCE has a positive impact on EPS (earnings per share) while CEE hurts ROI. In addition to this [23] selected 39 Iranian insurance companies from the study period 2005–2007 concluded that the value-added intellectual capital and its components has a positive impact on financial performance measured by ROA. [24] on the role of intellectual capital efficiency measured by Modified intellectual capital efficiency on the financial performance of selected 30 Indian knowledge-intensive (software and pharmaceutical) companies for the study period 2010–2014 suggested that the modified intellectual capital plays an important role in the value creation process. Additionally, the study concludes that the inclusion of relational capital in the value-added model extends the explanatory model and maximizes the financial performance of firms.

Further, a comparative study on the role of MVAIC and Pulic VAIC on companies’ performance by [32] from the study period 2000–2014 using panel data concludes that the modified VAIC model to some extent captures the structural capital efficiency of a firm more efficiently than the original model. Additionally, a study by [33] on intellectual capital and profitability in an emerging insurance market selected 36 life and non-life insurers in Ghana suggested that non-life insurers have higher intellectual capital performance than life insurers and there is a positive significant relation between intellectual capital and profitability. While HCE is the key driver of insurers’ intellectual capital performance.

An empirical study conducted by [25] in Pakistan insurance sectors on the effect of Intellectual Capital efficiency measured by [34] VAIC^™^ on the financial performance of insurance sectors measured by ROA, ROI, and EPS stated that VAIC^™^ has a positive and significant impact on ROA, EPS, and ROI, while among components of intellectual capital efficiency HCE and SCE have a positive significant impact on financial performance (ROI, ROA, and EPS). On the other hand, CEE has a negative significant impact on ROI.

Furthermore, a study conducted on the relationship between modified value-added intellectual capital efficiency and traditional financial performance (market to book value, ROA, and ROE) of Indonesian 50 bigger companies during the study period 2007–2014 by [35], stated that MVAIC^™^ positive influence on current and future financial performance of firms. Further, the finding proposed that the firm’s financial performance. The coefficient can be used to predict a future firm’s financial performance.

Moreover, an empirical study conducted by [36], on the effect of intellectual capital on the financial performance of Islamic insurance sectors. The study used the Pulic value-added intellectual coefficient to measure intellectual capital. The study used RBC and ROA to measure the financial performance of selected insurance stores. The study used 7 insurance sectors through analyzing least square. Finally, the finding of the study shows that Intellectual capital has a positive significant relation with financial performance.

## Research methodology

### Research design and approach

To achieve the objective, the research employed an explanatory research design. This design involved collecting secondary data through document analysis in an ex-post, longitudinal time dimension. The study aimed to determine whether the efficiency of insurance companies and their components contribute to performance. The research adopted a deductive method of inquiry with a quantitative research approach. The rationale for using this approach lies in its ability to analyze numerical data using statistical tests, such as inferential and descriptive statistics. Additionally, the study examined the collinear relationship between independent and dependent variables to conclude the impact of the independent variables on the dependent variables.

### Data source and method of data collection

To accomplish the study’s objective, the researchers relied on secondary data collected from the period spanning 2012 to 2022. The data were sourced from the annual financial statements of insurance companies, specifically obtained from the National Bank of Ethiopia (NBE). In Ethiopia, there are currently 17 recognized insurance companies. For this study, a purposive selection approach was employed, resulting in the inclusion of audited financial statements from 14 insurance companies. These statements covered a continuous period of ten years, from 2012 to 2022.

### Model specification

To achieve the stated objectives this study employed OLS Panel data multiple regressions since panel data offer a quick and affordable way to monitor how people’s opinions and behaviors change over time, and it can improve model parameter inference accuracy while simplifying computation and statistical inference [37, 38]. Hence, the following regression model was used by the researchers with some modifications depending on prior studies on the issue under investigation such as [24, 31, 35, 39–43].

To examine the effect of modified value-added intellectual capital introduced [19] by and later developed by [44] and its components (HCE, SCE, RCE, and CEE) on financial performance of insurance companies measured by ROA the researchers used Eqs 1 and 2 respectively.


$$ROA_{it}=\beta_{0}+\beta_{1}MVAIC{™}_{it}+u_{it}$$ (1)

$$ROA_{it}=\beta_{0}+\beta_{1}HCE_{it}+\beta_{2}SCE_{it}+\beta_{3}CEE_{it}+u_{it}$$ (2)

**Where**:

ROA_it_ is = Return on assets for insurance “i" while the periods in "t".

MVAIC^™^_it_ = Modified value-added intellectual capital efficiency coefficient

HCE = Human capital efficiency

CEE = Capital employed efficiency

RCE = Relational capital efficiency

SCE = Structural capital efficiency β_0_ = constant

U_it_ = error component for insurance “i” at time t and it assumed to have zero mean u_it_ = 0

β1, 2, 3and 4 are parameters to be estimated

i = insurance companies

t = periods

## Variables description and measurements

### Measurement of dependent variable: Financial performance

In this study, return on assets is used as a measurement of financial performance. The reason for choosing ROA as the vital proxy for insurance companies’ profitability instead of the alternative return on equity (ROE) is that an analysis of ROE disregards financial leverage and its risks [45]. ROA indicates how profitable a company is of its total assets. It gives an idea of how efficiently the management uses assets to generate revenue. ROA = Net income/Total asset [46].

### Measurement of the independent variable: The modified intellectual capital efficiency and its components

To examine the effect of intellectual capital efficiency on the financial performance of insurance companies, the researchers used the modified value-added intellectual coefficient (MVAIC^™^), introduced by [34], and developed by [44] to measure independent variables rather than VAIC, since, the VAIC has limitation. For instance, it focused on the efficiency of capital investment of firms and efficiency of labor rather than IC efficiency. The VAIC ignores relation capital [39] and [40], In comparison to [34], the extended/modified value add intellectual capital efficiency provides a more accurate measurement. The value and knowledge from corporate networks among clients, vendors, distributors, rivals, and all other associated parties are contained in RC. However, according to [47], customer interactions are seen as the most crucial element.

**Step- 1: Calculation of Value Added (VA**_**it**_**) for all stakeholders**
$${\text{VA}}_{\text{it}}={\text{OUTPUT}}_{\text{it}}-{\text{INPUT}}_{\text{it}}$$
  The Value added by the firm for a given period “t” can be calculated by rearranging the profit equation as
R=S−B−DP−W−I−DD−T (3)
  Where R = change in retained earning
  S = sales revenue or premium for insurance companies
  B = bought-in materials & services–Administrative expenses
  DP = Depreciation W = Wages I = Interests DD = Dividends T = Taxes
  The Eq 3 can be rearranged as,
S−B=DP+W+I+DD+T+R (4)
S−B−DP=W+I+DD+T+R (5)
  Eq 4 is the gross-value-added approach, whereas Eq 5 is the net value-added approach. The left-hand side and right-hand side of Eq 5 calculate the Value added, and the distribution of the Value created by firms, including employees, debt-holders, stockholders, and government respectively. Value added is created by the organization during a particular financial year. DD plus R is equal to net income [48]. Hence, value added can be calculated as (Eq 6).
VA=S−B−DP=W+I+T+NI (6)
  Where: NI is after-tax income
**Step- 2: Calculation of value-added capital employed coefficient (VACE**_**it**_**)**
  Capital employed is the book value of the net asset of a firm [19]. This component of the value-added intellectual coefficient (VAIC^™^) includes physical and financial capital [7, 49]. It measures the Value of assets that contribute to a company’s ability to generate revenue, also known as operating assets. So Value added by capital employed coefficient it (VACE_it_).
$$\text{Capital}\;\text{employed}\left(\text{CE}\right)=\text{Total}\;\text{asset}-\text{Intangible}\;\text{asset}$$
  VACE = is the Value created by one unit of capital employed during period t.
**Step 3: calculation of value added human capital coefficient (VAHC**_**it**_**).**
  The value-added human capital coefficient is calculated by treating all expenses for employees as investments and no longer treated as costs; human capital is an investment in human capital during the period or total salary and wage, including all incentives.
$${\text{HC}}_{\text{it}}=\text{Investment}\;\text{in}\;\text{Human}\;\text{Capital}\;\text{during}\;\text{the}\;\text{t}\;\text{period}\;\left(\text{total}\;\text{salary}\;\text{and}\;\text{wage}\;\text{including}\;\text{all}\;\text{incentives}\right)$$
  VAHC_it_ Value added by one unit of human capital invested during the period of t.
**Step 4: calculation of value-added structural capital coefficient (STVA**_**it**_**).**
  When HC increases, the SC decreases; they are opposite. Based on this STVA_it_ is calculated as:
$${\text{SC}}_{\text{it}}={\text{VA}}_{\text{it}}-{\text{HC}}_{\text{it}}$$
  So, STVA shows the proportion of total VA accounted by structural capital.
**Step 5: calculation of value added relational capital coefficient (RVA**_**it**_**).**
  Relational Capital Efficiency (RCE), the third element of IC, is added in this MVAIC by [35] and it serves as an example of the effectiveness of relational aspect investment. Marketing expenses serve as a stand-in for relational capital, RC was added as a new IC component in a modified VAIC model. Expenses related to marketing, selling, and advertising were used as a stand-in for RC. The ratio of marketing, selling, and advertising costs to VA served as the benchmark for relational capital efficiency (RCE). Additionally, it has been confirmed by [31, 42, 50, 51] that the VAIC model that has been modified to include RC is more accurate than the original VAIC model at measuring IC. All marketing and sales promotion expenses are viewed as investments in capital to improve the organization’s external relationships (24). Relation capital is regarded as a component of intellectual capital and RCE = RC/VA RC are marketing costs [51],
**Step- 5 Calculation of modified value-added intellectual coefficient (MVAIC**_**it**_**)**
$$MVAIC_{it}=VAHC_{it}+VACE_{it}+STVA_{it}+RCVA_{it}$$
  Finally, value added (VA) = Net income + Employee salary and benefit + Interest + Tax
$$\text{Intellectual}\;\text{Capital}\;\text{efficiency}\;\left(\text{ICE}\right)=\text{HCE}+\text{SCE}$$
$$\text{Modified}\;\text{Value}\;\text{added}\;\text{intellectual}\;\text{coefficient}\;\text{it}\;\left(\text{MVAIC}™\right)={\text{ICE}}_{\text{it}}+{\text{CEE}}_{\text{it}}{\text{RCE}}_{\text{it}}$$
  [44]
  MVAIC^™^ indicates the intellectual ability of value creation and how much new Value has been created per Invested monetary unit in each resource [52] The higher the coefficient the, better the company’s intellectual capital [19].

### Data analysis techniques

This study used descriptive and inferential statistics. The study used STATA software version 15.0 outputs to determine the relationship between the dependent and independent variables. Econometric model specification tests including the F-test, Breusch and Pagan Lagrange Multiplier test, and Hausman test were used to select the best-suited model among the pooled regression model, fixed effect model, and random effect model. In the same fashion, diagnostic tests for the classical linear regression model assumptions were carried out, and the random effect model was selected for analysis and presentation.

### Variables description and measurements

In the following table (Table 1) the study variables and their corresponding measurements are presented.

**Table 1:** Summary of measurement of variables.

No	Variables		Symbol	Measurement	Exp. sign
1	Dependent	Return on asset	ROA	Net income /Total asset	
2	Independent	Modified Value-added intellectual coefficient	MVAIC^™^	ICE + CEE (HCE + SCE + CEE+RCE)	**+**
		Human capital efficiency	HCE	HCE = VA /HC	**+**
		Structural capital efficiency	SCE	SC = VA-HC SCE = SC /VA	**+**
		Capital employed efficiency	CEE	CE = TA-IA CEE = VA/CE	**+**
		Relational Capital Efficiency	RCE	RCE = Marketing and advertising expenses/VA	**+**

**Source:** by the authors based on empirical review.

**Note:** Symbols (+ or -) in the above table the sign used to positive (+) and negative (-) expected sign

### Data presentation and discussions

#### Intellectual capital performance of selected Ethiopian insurance companies

The total modified value-added intellectual coefficient MVAIC^™^ developed by [44], used as a performance indicator, provides an understanding of total value creation through companies’ resources. Therefore, a higher value-added intellectual capital coefficient shows more significant value creation using an insurance company’s resources. At the same time, a lower coefficient indicates lower value creation.

As depicted in Table 2 empirical results indicate that HCE is the leading value creator, and it strongly contributes to value creation, followed by SCE, CEE, and RCE for all selected insurance sectors. The dominance of HCE is not surprising since the insurance sector is a service where customer services rely heavily on human capital [25]. In this study, by using the MVAIC^™^, the researchers attempted to rank selected insurance companies in Ethiopia during the study period 2012–2022. According to [19, 53, 54], firms scored above 5.0 and above show top performer; between 4.0 and 5.0 are considered to possess good performance, and between 2.5 and 4.0 represent common (this is the lowest level of efficiency which should be fulfilled to ensure safe workplace and safety) and below 2.5 bad performers. As duplicated in Table 2, the result shows that the intellectual performance of Ethiopian insurance sectors is entirely satisfactory.

**Table 2:** Average intellectual capital performances of selected Ethiopian insurance companies during the period 2012–2022.

No	Insurance companies in Ethiopia	HCE	SCE	RCE	CEE	MVAIC^™^	Rank
1	Ethiopia insurance corporation	4.66	0.85	0.01	0.78	6.30	Top
2	Awash Insurance Company S.C	5.52	0.72	0.01	0.70	6.95	Top
3	Global Insurance Company S.C	5.75	0.79	0.00	0.44	6.98	Top
4	Nile Insurance Company S.C	4.56	0.89	0.01	0.37	5.83	Top
5	Nice insurance company S.C	5.41	0.87	0.00	0.15	6.43	Top
6	Africa Insurance Company S.C	4.16	0.77	0.01	0.08	5.01	Top
7	NIB Insurance Company S.C	3.96	0.69	0.04	0.27	4.96	Good
8	Nyala insurance company S.C	7.00	0.95	0.01	0.18	8.14	Top
9	Unic Insurance Company S.C	6.34	0.88	0.01	0.17	7.39	Top
10	Oromia Insurance Company S.C	6.92	0.92	0.01	0.07	7.93	Top
11	Lion Insurance Company S.C	5.43	0.83	0.01	0.32	6.59	Top
12	Abay Insurance Company S.C	5.43	0.83	0.01	0.32	6.59	Top
13	Berhan Insurance Company S.C	5.50	0.83	0.01	0.28	6.62	Top
14	Tsehay insurance company S.C	5.49	0.84	0.01	0.24	6.59	Top

Source: Own analysis (2023)

#### Empirical results of regression analysis and discussions

Before proceeding to the result of regression analysis, diagnostic tests were carried out accordingly to check whether the data in the model fit or not the basic classical linear regression model assumptions. From the many of classical linear regression model assumptions that need to be satisfied Zero mean value of disturbance, Normality of residuals, No perfect multicollinearity, Equal variance of disturbance, and No autocorrelation between the disturbances assumptions were tested in this study accordingly.

After conducting a Diagnostic test for classical linear regression model (CLRM) assumptions, the study used the F-test, Breusch, Pagan Lagrange Multiplier, and Hausman-test to choose appropriate panel data estimation (see Tables 3–6 in S1 Appendix). Finally, the best model used in this study was the random effect regression model for both regression Eqs 1 and 2 (see Tables 3 and 4).

**Table 3:** Random effect regression result for Eq 1.

Random-effects GLS regression		Number of obs and group			[154, 14]	
Group variable: CompCod		Wald chi2(1) and Prob > chi2 =			[7.20,0.0073]	
ROA	Coef.	Std. Err	z	P>z	[95% Conf. Interval]	
MAVIC	.0146434	.0054568	2.68	0.007**	.0039482	.0253386
_cons	.0749889	.0382638	1.96	0.050	-6.70e-06	.1499845
sigma_u .06427725		sigma_e .13819492			rho .17785932 (fraction of variance due to u_i)	

Source: Own analysis (2023)

**Table 4:** Random effect regression result for Eq 2.

Random-effects GLS regression		Several obs. and group			[154, 14]	
Group variable: CompCod		Wald chi2(1) and Prob > chi2 =			[19.14, 0.0007]	
ROA	Coef.	Std. Err	z	P>z	[95% Conf. Interval]	
HCE	.0120003	.0057693	2.08	0.038 *	.0006926	.0233079
SCE	.0265981	.0303001	0.88	0.380	-.032789	.0859852
RCE	.027827	.1767915	0.16	0.875	-.318678	.3743321
CEE	.1451073	.0389081	3.73	0.000**	.0688489	.2213657
_cons	.0420533	.0425207	0.99	0.323	-.0412858	.1253923
sigma_u .06640915		sigma_e 13430059			rho .19647211 (fraction of variance due to u_i)	

Source: Own analysis (2023)

## Discussions

The overall aim of this study was to explore the effect of intellectual capital efficiency and its components on financial performance. The major findings of the study are presented as follows:

Firstly, the study examines the relationship between intellectual capital efficiency and financial performance. The modified value-added intellectual coefficient regression coefficient has a positive and significant impact on the financial performance of insurance companies in Ethiopia. The result of the study is consistent with [31, 24, 23, 35, 36]. According to [35] MVAIC has a favorable impact on both recent and upcoming financial performance. Consequently, financial performance may be predicted using MVAIC. The findings deepen our understanding of how intellectual capital contributes to corporate value creation and the development of long-term competitive advantages for businesses in emerging nations. In line with this finding, [55] human capital improves the financial performance of insurance sectors. Insurance sectors need human capital with dedicated skills and talent. Employee training related to intellectual capital is a prominent investment. The higher cost incurred for employee training escalates talent and skill and improves the performance of firms. Further, the study suggested that insurance firms require specialists like insurance firm experts.

On the contrary, a study conducted by [28, 56, 57] on companies operating in South Africa revealed that IC has no significant impact on a firm’s performance. Furthermore, [58] stated that ICE measured by the Public VAIC has no significant impact on the financial performance of Greek firms. Further, the researchers provide a cornerstone justification for those who stated that VAIC^™^ had not affected the financial performance of firms. The reason might be developing countries’ lack of advanced accounting practice and mature financial structure to fit the requirement of VAIC^™^, since the relationship between VAIC^™^ and financial performance with developing nations like Tanzania, Greece, Iran, and South Africa.

Secondly, the study was deemed to analyze the relationship between human capital efficiency and financial performance. The regression coefficient of human capital efficiency is positive and significant at a 5% significance level. The study is consistent with [25, 23, 58, 59] which stated that human capital (skill, knowledge, and employee competence) significantly impacts firms’ financial performance. Since individuals always own human capital. Firms better use their human capital to pursue financial performance and extended-lasting survival. Further, it indicates that better-trained and skilled employees tend to add value to the insurance company through profit. Well-equipped and better-trained employees reduce expenses and maximize profitability. Indeed, the result of this study proves that intellectual capital is the knowledge that affords information about a firm’s soft asset and its value. Which may affect sustainability and contribute to achieving firms’ goals besides superior competitive advantage [55]. If insurance companies want to stay ahead of the market, they must make sure that their human capital is more knowledgeable about their core and emerging business concerns [60].

On the contrary to this finding, [28, 30, 49, 61] stated that human capital significantly negatively impacts firms’ financial performance. Human capital embraces individual economic value, core competence, and financial performance [5]. Since human capital requires support from structural capital [20, 62] suggested that HCE harms the financial performance of Malaysia’s financial sector as a result of the measurement of human capital through Pulic VAIC^™^ might be unsound. Since it does not measure the actual value of the human resource, it measures the value added per $ salary and wage; further, HC might be used for other agenda that does not support the organizational goal.

Finally, the study examines the relationship between capital employed efficiency and financial performance. In this study, capital employed efficiency measured by value-added divided by firms’ physical and financial assets has a positive and significant impact on an insurance company’s financial performance. In line with this study, [29, 28]. [63] suggested that capital-employed efficiency has a significant impact on a firm’s financial performance. Contrary to this study, [9] CEE has a negative significant impact on the financial performance of Pakistan firms [64] stated that capital-employed efficiency negatively impacts the financial performance of 300 companies operating in the UK. The negative sign indicates that the firm’s physical and financial assets may generate additional expenses like electricity expenses for operating machines, which are part of the capital employed.

To sum up, CEE which includes a firm’s tangible and financial assets has a positive significant impact on an insurance firm’s financial performance. The positive sign signposts, that the Ethiopian insurance sector is still generating revenue through using tangible and financial assets.

## Conclusion

The result indicates that IC measured by modified valued added intellectual coefficient has a significant positive impact on the financial performance of insurance companies. This study proves that IC is not the only central trading tool; it is the backbone of the insurance sector. The result underlines the role of IC in accelerating financial performance. Therefore, insurance sectors should nourish the concept and application of intellectual capital to achieve financial performance.

In this study, the researcher attempts to rank the capacity of ICE measured by the MVAIC^™^ of insurance sectors in Ethiopia based on [19, 53, 54], level of measuring IE. However, the insurance sector’s intellectual efficiency is very satisfactory since the lowest efficiency level is 4.96. It indicates that selected insurance companies are ensuring a safe workplace and safety.

Human capital has a positive impact on the financial performance of insurance companies. The study’s result is not surprising since insurance sectors are knowledge-intensive sectors. Where their customer services rely heavily on human capital, this notice, skill, knowledge, and employee competence significantly contribute to financial performance. Insurance sectors better use their human capital to achieve financial performance and long-lasting survival for insurance companies in Ethiopia. Better-trained and skilled employees tend to add value to the insurance companies in the form of profit. Through reducing expenses and maximizing profitability. CEE has a significant impact on financial performance. This shows insurance companies still generate revenue through using tangible and financial assets.

Insurance companies that can better use their human capital tend to survive and achieve their goal. Hence, it recommended that insurance sectors identify and hire critical people, further provide continuous training programs to exalter managers’ and employees’ performance, and ensure accessibility of human resources in the right quality and quantity.

### Theoretical implication

The findings of this study contribute to the theoretical understanding of the relationship between intellectual capital efficiency and financial performance in the context of insurance companies. By adopting the Modified value-added intellectual coefficient as a measure of intellectual capital efficiency, the study provides a novel approach to assessing the impact of intellectual capital on financial performance. The positive and significant association between valued added intellectual capital, human capital efficiency, and capital employed efficiency with financial performance suggests that investments in these components of intellectual capital can lead to improved financial outcomes. On the other hand, the lack of a significant contribution from relational capital efficiency and structural capital efficiency highlights the need for further investigation and possible revisions in the existing theoretical frameworks relating to these specific components of intellectual capital in the insurance industry.

### Practical implication

The practical implications of this study are particularly relevant for insurance companies in Ethiopia. The results underscore the importance of focusing on and enhancing value-added intellectual capital, human capital efficiency, and capital employed efficiency to achieve better financial performance. Insurance firms should consider investing in employee training and development to improve human capital efficiency, optimizing the utilization of capital to increase capital employed efficiency, and fostering a culture of innovation and knowledge sharing to enhance valued added intellectual capital. By doing so, insurance companies can position themselves for greater financial success and competitive advantage in the market. Moreover, the study’s adoption of panel data analysis and econometric modeling provides a robust methodology that can be applied in similar research contexts to assess the relationship between intellectual capital and financial performance in other industries or regions.

### Managerial implication

Managers and executives in the insurance industry can derive valuable insights from this study to inform their strategic decision-making. The positive and significant relationship between valued added intellectual capital and financial performance suggests that managers should actively identify, measure, and manage their company’s intellectual capital. They should implement strategies to enhance the efficiency of human capital and ensure optimal utilization of capital resources. These strategies may include talent development programs, performance-based incentives, and effective risk management practices. Furthermore, the lack of a significant contribution from relational capital efficiency and structural capital efficiency indicates that managers may need to reevaluate their approaches to managing these aspects of intellectual capital. Collaboration and relationship-building with stakeholders and leveraging technology and organizational processes to improve structural capital efficiency could be avenues for improvement.

#### Implications for future research

Finally, the study proposed that future research should investigate the effect of intellectual capital efficiency on the financial performance of financial and non-financial firms in knowledge-intensive industries like telecommunications, trading, and service firms.

## Supporting information

S1 Appendix (DOCX)
