# Supplementary material for: Does intellectual capital efficiency measured by modified value-added intellectual coefficient affect the financial performance of insurance companies in Ethiopia?
Source: PLoS One. 2024 Jan 19;19(1):e0295321. doi: 10.1371/journal.pone.0295321 (PMC10798503; doi:10.1371/journal.pone.0295321)
Supplement: S1 Appendix — (DOCX) [file pone.0295321.s001.docx]

**Appendix: S1. Supporting Information**

**Table 1. Correlation between study variables**

| Variables | HCE | SCE | RCE | CEE | ROA | VAICTM |
| --- | --- | --- | --- | --- | --- | --- |
| HCE | 1.0000 |  |  |  |  |  |
| SCE | 0.3051 | 1.0000 |  |  |  |  |
| RCE | -0.3187 | -0.2787 | 1.0000 |  |  |  |
| CEE | -0.0181 | -0.0501 | -0.0935 | 1.0000 |  |  |
| ROA | 0.1845 | 0.1324 | -0.1297 | 0.2714 | 1.0000 |  |
| VAICTM | 0.9804 | 0.4344 | -0.3288 | 0.1094 | 0.2288 | 1.0000 |

**Table 2. Multicoinerity test and VIF**

| **Variables** | **VIF** | **1/VIF** |
| --- | --- | --- |
| HCE | 1.18 | 0.846770 |
| RCE | 1.18 | 0.850389 |
| SCE | 1.15 | 0.866035 |
| CEE | 1.02 | 0.983920 |
| Mean VIF | 1.13 |  |

**Table 3. Breusch and pagan lagrangian multiplier test (Equation 1)**

| ROA[CompCod,t]=xb + u[CompCod] e(Comp Cod,t] | | | |
| --- | --- | --- | --- |
| Estimated results | |  |  |
|  | var | Sd=sqrt(Var) |  |
| ROA | .0236512 | .1527898 |  |
| e | .0190978 | .1281949 |  |
| u | .0041316 | .0642772 |  |
| Test Var(u)=0 | | |  |
|  | Chibar2(01)=16.97 | |  |
|  | Prob>chibar2=0.0000 | |  |

**Table 4. Hausman test (Equation 1)**

|  | coefficients | | | |
| --- | --- | --- | --- | --- |
|  | (b) fixed | (B) random | (b-B)difference | Sqt(dig(v_b-v_b)) s.E. |
| MVAIC | 0.147478 | 0.0146434 | 0.0001044 | .0025529 |

b=consistent under HO and obtained from Xtreg

B=inconsistent under Ha, efficient under Ho; obtained from Xtreg

Test : Ho: difference in coefficients are systematic

chi2(1) = (b-B) ‘ [(v_b-v_B) < (-1) ] (b-B)

= 0.00

Prob>chi2 = 0.9674

**Table 5. Breusch and pagan lagrangian multiplier test (Equation 2)**

| Breusch and pagan lagrangian multiplier test for random effect | | | |
| --- | --- | --- | --- |
| ROA[CompCod,t]=xb + u[CompCod] e(Comp Cod,t] | | | |
| Estimated results | |  |  |
|  | var | Sd=sqrt(Var) |  |
| ROA | .0236512 | .1537898 |  |
| e | .0180366 | .1343006 |  |
| u | .0044102 | .0664091 |  |
| Test Var(u)=0 | | |  |
|  | Chibar2(01)=16.35 | |  |
|  | Prob>chibar2=0.0000 | |  |

**Table 6. Hausman test (Equation 2)**

|  | coefficients | | | |
| --- | --- | --- | --- | --- |
|  | (b) fixed | (B) random | (b-B)difference | Sqt(dig(v_b-v_b)) s.E. |
| HCE | .125068 | .0120003 | .0005065 | .0022325 |
| SCE | .0223486 | .0265981 | -.0042495 | .00605 |
| RCE | .0743157 | .027827 | .0464887 | .0575234 |
| CEE | .1557565 | .1451073 | .0106492 | .0173601 |
| CEE |  |  |  |  |

b=consistent under HO and obtained from Xtreg

B=inconsistent under Ha, efficient under Ho; obtained from Xtreg

Test : Ho: difference in coefficients are systematic

chi2(1) = (b-B) ‘ [(v_b-v_B) < (-1) ] (b-B)

= 1.83

Prob>chi2 = 0.7668
